# Application of a risk-based standardized animal biomonitoring approach to contaminated sites

**DOI:** 10.1007/s10661-019-7653-3

**Published:** 2019-07-30

**Authors:** Paola Scaramozzino, Sabrina Battisti, Rosanna Desiato, Marco Tamba, Giorgio Fedrizzi, Alessandro Ubaldi, Bruno Neri, Maria Cesarina Abete, Giuseppe Ru

**Affiliations:** 1 Istituto Zooprofilattico Sperimentale del Lazio e della Toscana “M. Aleandri”, Via Appia Nuova, 1411 Rome, Italy; 2 Istituto Zooprofilattico Sperimentale del Piemonte, Liguria e Valle d’Aosta, Via Bologna 148, 10154 Torino, Italy; 3 Istituto Zooprofilattico Sperimentale della Lombardia e dell’Emilia Romagna “Bruno Ubertini”, Via Bianchi, 9, -25124 Brescia, Italy

**Keywords:** Biomonitoring, Animal, Environment, Health, Pollution, Contaminated sites

## Abstract

Biomonitoring has been used to disclose the public health impact of contaminated sites. This study aimed at setting up good practices to apply biomonitoring targeting animal matrixes to design risk-based surveillance and exposure assessment plans. A nine-step protocol targeting farmed animals was devised and tested in three case study areas including (1) a waste dump, (2) a waste incinerator, and (3) a secondary aluminum smelter. Between 2010 and 2012, in each study area, the following 9-step best practices were applied: hazard identification, GIS project creation, risk area delimitation, control area selection, receptors (livestock) identification, farms and matrixes selection, sampling study design, on-farm secondary sources exclusion, and statistical and geostatistical analysis. Dairy farms and free-range laying hens were the primary targets: eggs from both risk and control areas and milk from risk areas were sampled and submitted for detection of selected tracking contaminants compatible with the putative sources. Comparison data (risk vs. control) of heavy metal concentrations in eggs were available only for case study 2, whereas egg comparison data of persistent organic pollutants were available for all the risk-control pairs. After taking into account potential secondary sources, no concern from metals was arisen, whereas high concentrations of persistent organic pollutants were detected in all risk areas; however, only for the aluminum smelter case study, the contamination was broad and higher in the risk area compared with the control one. The protocol has proved to be easily applicable and flexible to varying contexts and able to provide helpful data to inform risk management decisions.

**Electronic supplementary material:**

The online version of this article (10.1007/s10661-019-7653-3) contains supplementary material, which is available to authorized users.

## **Introduction**

Chemical pollution of anthropic origin is recognized worldwide as one of the most challenging determinants of neoplastic and degenerative diseases in humans, especially in developed countries (Steenland and Savitz 1997).

Over the last decade, in Italy, several contaminated sites were identified and often originated legal cases in relation to the responsibility of the pollution. Several epidemiological studies were implemented in order to find evidence of an association between the exposure to contaminated sites and human health and to quantify that impact (Pirastu et al. 2014). Exposure assessment, a step of risk assessment (Codex Alimentarius Commission 2011), provides insights into such association although it may result in a difficult task because of several risk factors (e.g., lifestyle and smoking habits) acting as important confounders. In epidemiological studies, several approaches have been developed based on direct or indirect assessments such as personal exposure monitoring, proximity indicators, dispersion models, and environmental monitoring. Compared with simple indicators of exposure based on distance from the source, environmental monitoring provides data based on the measurement of contaminants in soil, air, and water; however, there is uncertainty on how much of the pollutants can overcome the body’s defenses and be absorbed. Moreover, the estimates of exposure derived from this indirect approach, at individual level, may be in part misclassified.

When affordable, human biomonitoring (i.e., the direct measurement of chemical contaminants in human specimens) may be used as a tool for internal dose measurement in contaminant exposure assessment (Hoek et al. 2018). The biomonitoring has the advantage to take into account at once all possible sources (food, water, air, and soil), all exposure pathways (respiratory, oral, and skin), and all individual influencing factors (diet, metabolism, age, pregnancy, etc.). It can be faster in ascertaining environmental pollution (early detection) since bioconcentration in organs and tissues makes animals more sensitive than environmental matrixes especially in areas where pollution has not yet raised to evidence. Biomonitoring can be used both for providing information on an emerging pollution outbreak and for monitoring the trend and the spread of contamination during and after the event, especially when mitigation measures have been undertaken. Human biomonitoring poses unique ethical challenges, such as those associated with specimen banking and results return (CSTE 2012).

With this regards, animal biomonitoring offers specific advantages when compared with human biomonitoring. As the animals are characterized by restricted daily mobility and are less likely affected by confounding factors, their biomonitoring can be more accurate and make the identification of the source of contamination easier (O’Brien et al. 1993; Van der Schalie et al. 1999). Moreover, in contaminated sites, the practice of feeding farm animals with local forages may arise in the population a fear of a hazard related to the food chain since it is known that one of the major ways of exposure to contaminants for humans is through diet (Ax et al. 2015; Traore et al. 2016; Malisch and Kotz 2014). Under these conditions, risk assessment and controls on food safety assume a pivotal role, either in case legal limits of the potentially involved contaminants being established or not. The study of the concentration of contaminants in specific animal tissues and the dynamics of excretions through biological liquids (i.e., milk) can then generate useful data about human exposure through the consumption of products of animal origin. Animal matrixes have the advantage of demonstrating the passage of pollution in the food chain and the actual risk for the population. Bioconcentration in organs and tissues makes biomonitoring more sensitive than environmental monitoring especially in areas where pollution has not yet raised to evidence. As an example, bovine milk is proved to be a good indicator of soil contamination by polychlorinated biphenyls (PCB’s) (Perugini et al. 2012). Finally, pre-existing sampling networks set up in the frame of food safety control campaings may offer the opportunity of carrying out animal biomonitoring exercises in a cost-effective way.

The present study, in agreement with the recommendation of WHO^1^ on health impact assessment, aimed at setting up good practices for animal biomonitoring to design a framework of risk-based surveillance (Scaramozzino et al. 2010) and animal exposure assessment plans in areas around contaminated sites.

For this purpose, we have developed good practice protocols and tested them through three case studies. The final objective is to provide evidence on how animal biomonitoring may contribute to generating essential information for an integrated environmental health risk assessment approach.

## Methods

In the following paragraphs, we will show the application of a general biomonitoring protocol using farmed animals in an environment with either an already identified source of pollution or a putative one. The general protocol is developed through nine standardized steps which are described below. The three case studies (study areas 1–3) differ in geographical location, source of the contaminants, natural environment, presence and typology of industrial plants, and livestock involved and for these reasons, the protocols were adapted to each single study setting.

### Methodological approach: a nine-step protocol

**Fig. 1:**
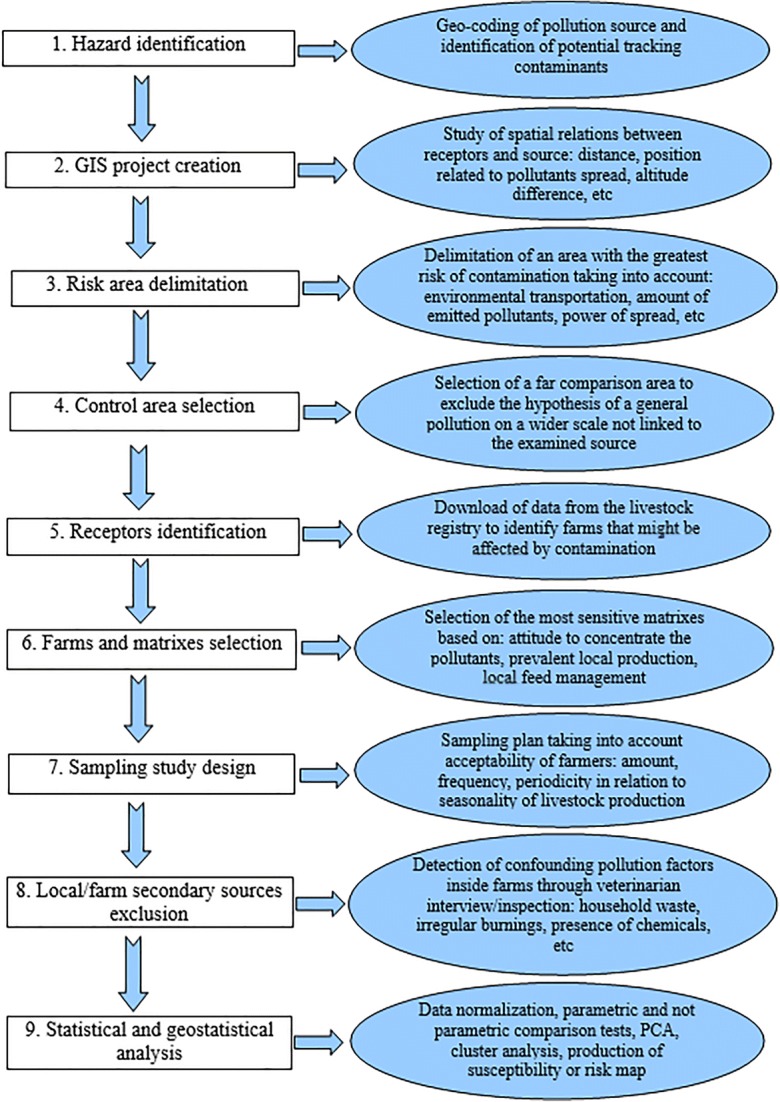
Flowchart listing the steps of the adopted protocol of risk-based animal biomonitoring

The adopted protocol (Fig. 1) consists of subsequent steps. They are listed below with a description of their practical application in the context of the selected case studies:

- Step 1: *Hazard identification*: i.e., identification and geocoding of the putative pollution source and hypothesis of potential tracking contaminants. In two of the case studies, there was no clear, preliminary evidence of generalized environmental pollution, but only sporadic records of metal presence in groundwater layer (study area 1) or detection of small amount of total dust, PM10, NO2, and traces of metals in the air (study area 2). In the third area (study area 3), the environmental contamination by persistent organic pollutants (POP’s) that originated from the smelter plant was already known from previous studies, and contaminants were detected in eggs from free-range hens reared in the surrounding farms. On the basis of the available knowledge for study area 3 and the plausible production of pollutants both in study areas 1 and 2 as an effect of combustion processes, POP’s^2^, known to produce bioaccumulation in farm animal tissues, was chosen in tracking contaminants for all study areas.
- Step 2: *GIS project creation*: using a geographic information system (GIS, i.e., a desktop mapping and spatial data analysis application), the spatial relations in terms of distance and position relative to the direction of the pollutant outflow between the source and the exposed farms (i.e., the receptors) needs to be studied and analyzed.
- Step 3: *Risk area delimitation:* in the absence of an available dispersion model or to integrate it, GIS instruments are used for the delimitation of the risk area around the pollution source. Given the characteristics of the putative sources of contamination of our case studies (releasing in top/subsoil for study area 1 and in the air for study areas 2 and 3 respectively), risk areas were of different sizes. We assumed that the spread of pollutants would cover a larger area in case of air emission because of the power of incinerator and the higher amount of delivered pollutants.
- Step 4: *Control area selection*: a control area is defined far from the pollution source to test the hypothesis that the contamination could be due to general pollution on a wider scale and not to the contaminated site under examination. In the present study, for study areas 1 and 2, the entire area outside the risk area, corresponding to the administrative boundary of the province, was chosen as the control area, after an ad hoc investigation to exclude the proximity of other known putative sources of the same contamination to the sampled farms. With regards to study area 3, the entire region was considered. Moreover, as described in Step 8, the potential for farm-level confounding factors was excluded through a dedicated investigation.
- Step 5: *Receptors identification*: i.e., identification of who might be affected by the contamination; for the purpose, we downloaded from the official livestock registry (National Animal Recording System, www.vetinfo.it) the data (i.e., location, species, breeding type, and herd size) of all the farms included respectively in the risk and control areas.
- Step 6: *Farms and matrixes selection*: Fit for purpose, criteria of farms/matrixes selection have to be defined: in our case, farms were selected according to the following criteria of eligibility: (i) animal tissue/product sensitivity (i.e., attitude to concentrate/accumulate pollutants), (ii) prevalent livestock production system, (iii) feed management (at least one item of the animal feed coming from local production), (iv) number of animals present in each farm, and (v) sampling cost. Dairy farms (with cattle or sheep) or free-range laying hens were selected as primary targets. Dairy farms with less than 10 heads were excluded because of their variability in number of reared animals and production over time; large, intensive farms with minimal interaction with the surrounding environment were also considered not eligible. Local veterinarians were interviewed to confirm the practice of farmers to feed animals with at least one item of local origin (fresh forage or hay) considering that feeding is the most important route of exposure for environmental pollution intake. Apart from the pathways of transfer/exposure, the additional selection criteria for the collection of the targeted sampling matrixes were: (i) inclusion of the same type of matrix in already existing monitoring plans implemented for reasons other than the scope of the study (i.e., surveillance plans to monitor chemical hazards in food), and (ii) easiness of sampling to optimize costs. Moreover, the importance of the selected matrixes in the human diet was considered. In control areas, the same criteria of eligibility (species, animal matrix, local feed origin) were used to select the farms for the collection of samples. The delimitations of both risk and control areas and farms to be enrolled have been defined through a GIS project (Step 3 above) and overlaying different layers (livestock registry, source points, and buffer layers). To avoid a potential role for confounding factors, some ad hoc investigations inside farms have been carried out (Step 8).
- Step 7: *Sampling study design*: preliminary frequency of sampling is defined. The acceptability by the farmers and the practical feasibility of the monitoring procedures needed to be considered. The frequency of sampling was established taking into account the seasonality of the animal productions (e.g., exclusion of dry period, in order to maximize the probability of catching all the milking animals or periods of seasonal laying). Each sample of milk was at least 500 ml, and each egg sample required 6 eggs.
- Step 8: *Local/farm secondary sources exclusion*: secondary sources of contamination, i.e., farm-specific and therefore not associated with the external common source of interest, might have confounded the cause-effect investigation (Hoogenboom et al. 2016): the potential confounding effect has to be addressed. A dedicated standardized questionnaire was administered to the farmers by trained veterinarians (ESM 1). Interviews were paralleled by visual inspection on the farm to detect any farm-specific potential secondary source. The following factors were considered: household waste, irregular/occasional burnings, percolation of stored chemical substances (oil, paints, and soap), proximity to high traffic roads, asphalt/bitumen presence on grazing soil, presence of plastic items in the animal recoveries, etc. In case of detection of contaminants, the analytical results coming from farms where confounding factors had been detected were carefully re-evaluated.
- Step 9: *Statistical and geostatistical analysis*: a range of statistical techniques needs to be considered. They aim at comparing risk vs. control areas (e.g., through parametric or non-parametric tests), at characterizing the profile of the contaminants detected (e.g., through Principal Component Analysis or Cluster Analysis) or at estimating the geographical distribution of the contamination (e.g., through regression models or interpolation techniques such as Kriging) to obtain risk maps (Battisti et al. 2013; Desiato et al. 2014).

### Study areas and period

The protocol for animal biomonitoring was applied according to common criteria, in different Italian contexts, focusing on the three main potential sources of environmental contamination: a waste dump, a waste incinerator, and a secondary aluminum smelter.

**Fig. 2:**
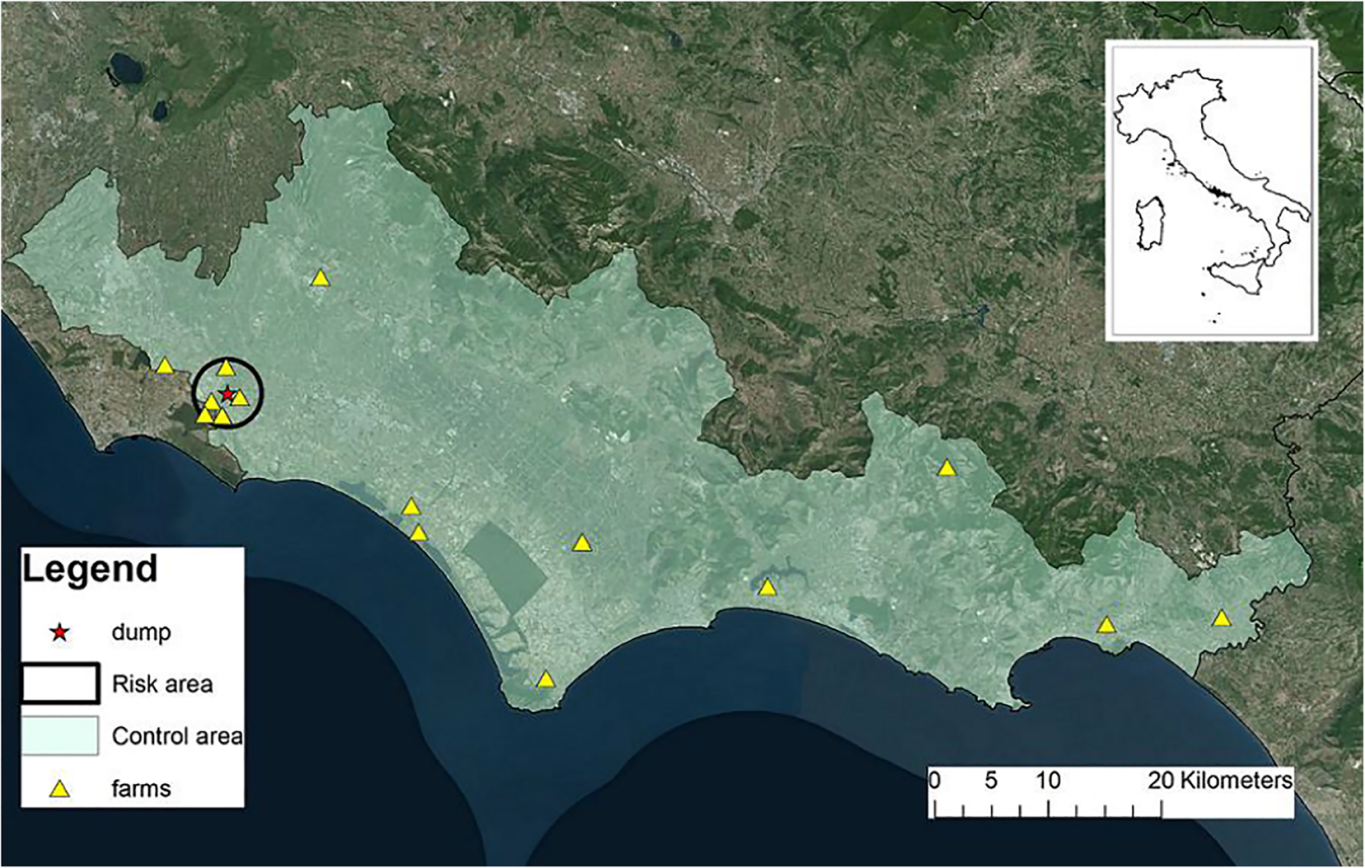
Latium study area (study area 1, Latina Province)

**Fig. 3:**
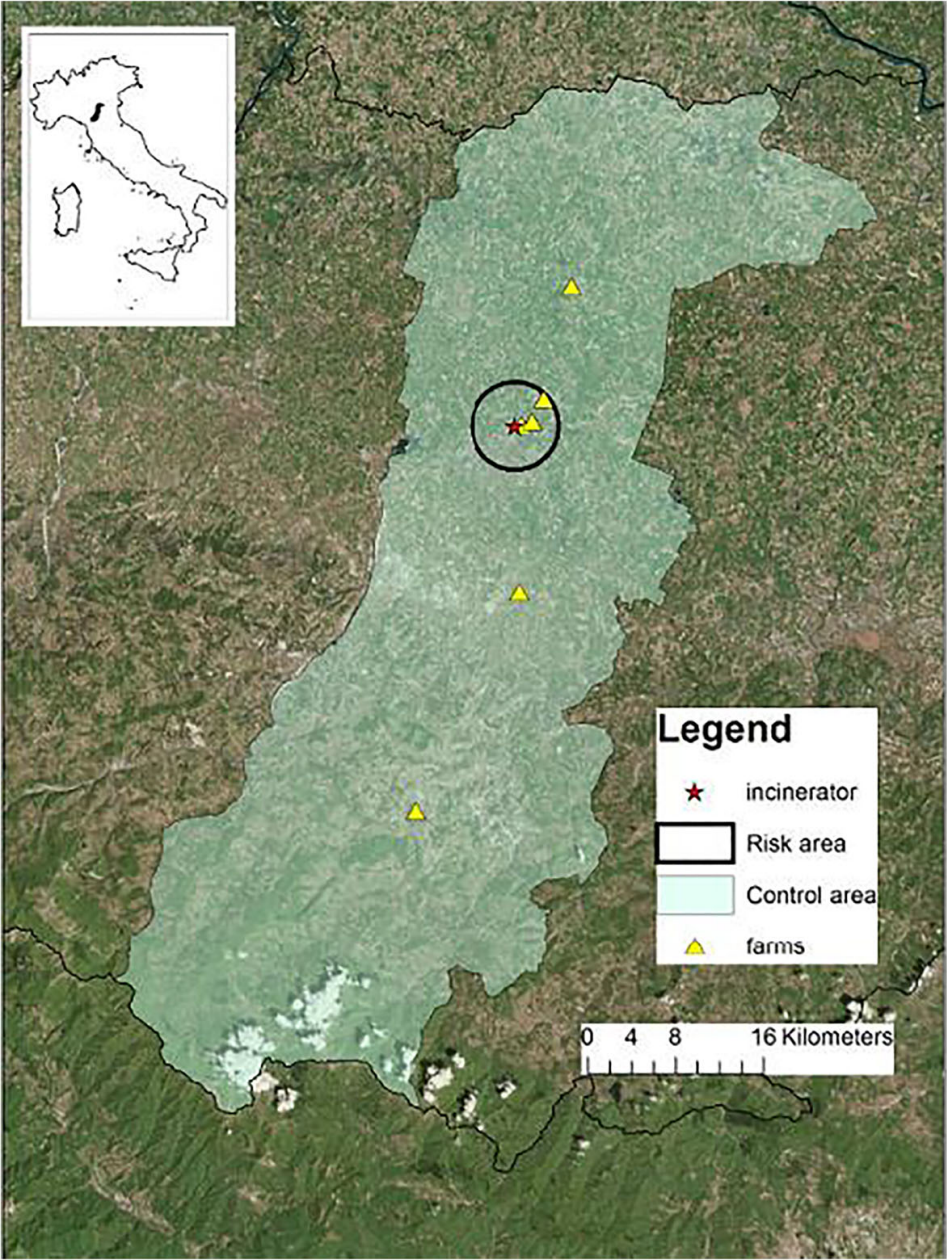
Emilia Romagna study area (study area 2, Modena Province)

**Fig. 4:**
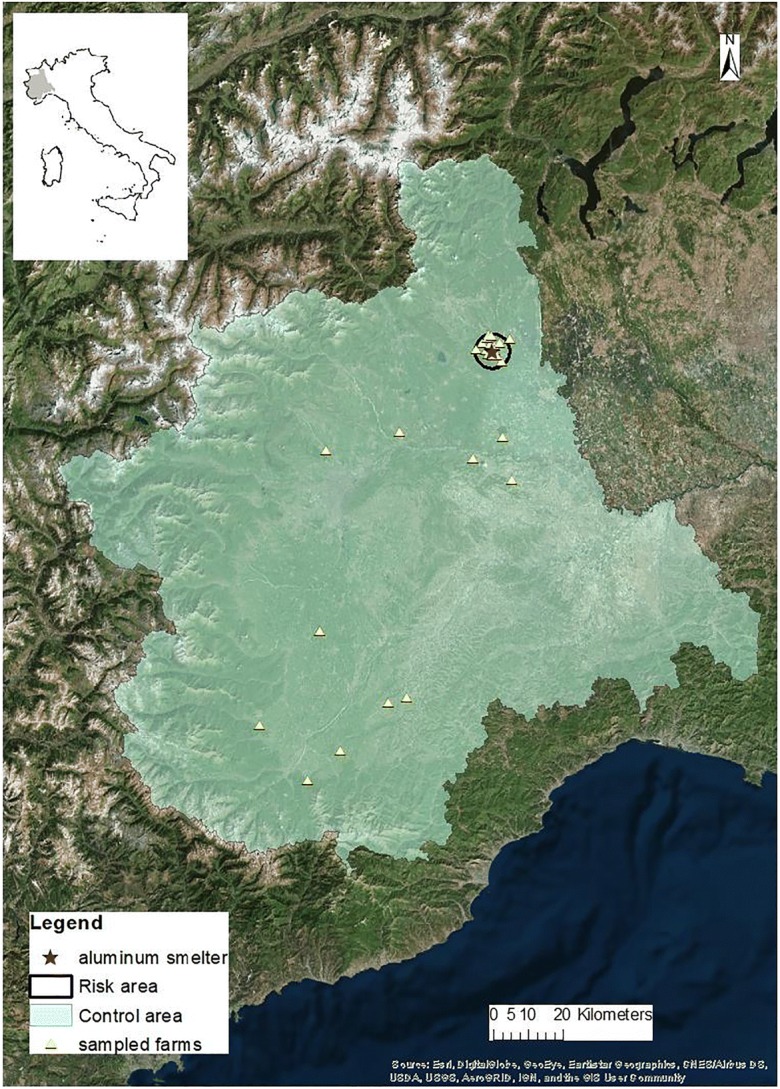
Piedmont study area (study area 3, Piedmont region)

The waste dump is located close to the city of Latina, in the Latium region (study area 1); the incinerator is located near the city of Modena, in Emilia Romagna (study area 2), a region vocated to the Parmigiano Reggiano production; the secondary aluminum smelter is located in an eastern area of Piedmont region where rice cultivation is the main economical activity (study area 3, all the region was considered). In Figs. 2, 3, and 4, the three areas are shown.

Whereas the waste dump is a potential source of pollutants in the soil due to percolation of liquid, both the waste incinerator and the aluminum smelter are air emitters due to industrial combustion processes. In the case of study area 3, the environmental contamination was already confirmed in previous studies (Colombo et al. 2011; Squadrone et al. 2015), whereas in the case of study areas 1 and 2, there was neither evidence nor anecdotal report about the presence of pollutants in water or soil which was of great concern among the people living in the surrounding areas.

A common sampling protocol was developed taking into account species and numbers of farmed animals, prevalent type of food production chain of animal origin, and geographical features such as distance from the source and orography of each area.

The biomonitoring was carried out between 2010 and 2012.

### Laboratory methods

Here we listed the contaminants and described the analytical chemical techniques used in our specific case studies:

- Polychlorinated dibenzo-p-dioxins (PCDDs), polychlorinated dibenzofurans (PCDFs), and dioxin-like polychlorinated biphenyls (dl-PCBs): all analytes were identified, confirmed, and quantified by gas chromatography with high-resolution mass spectrometry (GC–HRMS) and isotopic dilution technique, according to the EPA methods 1613 and 1668.
- Lead (Pb) (limit of detection (LOD) − limit of quantification (LOQ): 0.004–0.008 mg/kg), cadmium (Cd) (LOD − LOQ: 0.001–0.002 mg/kg), chromium (Cr) (LOD − LOQ: 0.003–0.006 mg/kg)), manganese (Mn) and arsenic (As) (LOD − LOQ: 0.01–0.02 mg/kg), and zinc (Zn) and nickel (Ni) (LOD − LOQ: 0.002–0.005 mg/kg): quantified by atomic absorption spectrophotometry with graphite furnace (GF-AAS)
- Mercury (Hg): (LOD − LOQ: 0.001–0.002 mg/kg) quantified by cold vapor atomic absorption spectrophotometry CV-AAS.

With regards to POP’s, all data were expressed as Toxic Equivalents (TEQs) since PCDD/Fs and dl-PCBs are usually present as complex mixtures containing several kinds of congeners. TEQ values are calculated using weighting factors expressing the toxicity of each individual PCDD/Fs and dl-PCBs congener compared to 2,3,7,8-TCDD, the most toxic congener, to which is assigned the arbitrary factor of 1. The weighting factor, termed toxic equivalent factor (TEF), is multiplied by the concentration of the individual congener to give a 2,3,7,8-TCDD equivalent toxicity. TEF values established by the World Health Organization in 2005 were used. The unit of measurement is respectively pg WHO-TEQ-2005/ g fat for PCDD / F and dl-PCB and mg/kg^a^ for heavy metals.

### Statistical methods

The GIS project creation and all the investigations based on spatial data were carried out by using ArcGis (10.3). With regards to the analytical results, after testing normality through the Shapiro-Wilk test, a two-sample Wilcoxon rank-sum (Mann-Whitney) test was used to detect significant differences in mean concentration between risk and control areas (*p* < 0.05). Finally, in study area 3, a linear regression model, fitting the logarithmic values of POP’s concentrations as dependent variable, was used to check if the degree of egg contamination was associated with the distance from the source. For the estimation of heavy metal average, the values below the limit of detection were considered equal to 0. All statistical analyses were carried out with the statistical software package Stata 14.1 (StataCorp. 2015. Stata: Release 14. Statistical Software. College Station, TX: StataCorp LLC).

## Results

### Development of the Sampling protocol

After the identification and the geocoding of the three putative sources (Step 1), the following POPs and heavy metals were chosen as tracking contaminants in milk and eggs (Table 1): polychlorinated dibenzo-p-dioxins, polychlorinated dibenzofurans, dioxin-like polychlorinated biphenyls, cadmium, chromium, mercury, lead, arsenic, zinc, manganese, and nickel.

**Table 1:** Group of substances analysed in different matrixes

Substance group	Matrix
Polychlorinated dibenzo-p-dioxins (PCDDs)	Milk, eggs
Polychlorinated dibenzofurans (PCDFs)	Milk, eggs
Dioxin-like polychlorinated biphenyls (dl-PCBs)	Milk, eggs
Cd, Cr, Hg, Pb, As, Zn, Mn, Ni	Milk, eggs^a^

^a^Analysed only in Emilia Romagna case (study area 2)

The choice of POPs or metals as tracking contaminants was based on the following criteria: (i) evidence already available for study area 3, (ii) knowledge of the production processes in the other two study areas, and (iii) the toxicological risk for human health. In study areas 1 and 2, metals were also analyzed because of their suspected presence (study area 2) or the already existing evidence (study area 1) of environmental contamination. In the three areas irrespectively of the different pathways through which pollutants are dispersed in the environment, we have hypothesized that the main pathway of exposure for the animals was through soil and water (Raj et al. 2006; Mamontova et al. 2007; Lake et al. 2005). In the absence of available dispersion models, we chose to define risk areas (Steps 2 and 3) as circular respect to the source in consideration of a hypothetical isotropic diffusion of contamination from source site. By means of a GIS software, three circular buffers of 3-, 4-, and 5-km radius were chosen as risk areas around the sources of study areas 1, 2, and 3 respectively. The size of the risk areas 2 and 3 are larger because of aerial dispersion of the pollutants and geographical peculiarities.

Dairy cattle, grazing sheep, and free-range laying hens were identified as main receptors and therefore considered eligible and targeted for sampling (Step 5). Five sheep/goat and 3 cattle farms were sampled in risk area 1, 3 cattle farms in risk area 2, 3 cattle farms plus 15 farms with laying hens in risk area 3 (Steps 3, 5, and 6). Within a period varying from 2 to 6 months, in each risk area, matrixes were sampled twice to account for possible seasonal variations in milk/eggs production (Step 7). A total of 24 milk and 55 egg samples were analyzed.

In control areas 1, 2, and 3 (Steps 4, 5, and 6) for both cost-saving and higher sensitivity toward target substances, only eggs from 10, 3, and 13 free-range laying hens farms respectively were sampled once during the entire study period (Step 7).

A standardized questionnaire (ESM 1) was prepared and used on the spot by a veterinarian to exclude farm secondary sources of pollution as provided by Step 8.

### Analytical results

Heavy metal mean values (15 milk samples in risk area 1 and 6 in risk area 2) (ESM 2) were 3.55 mg/kg for Zn (risk area 2), 0.0042 and 0.006 for Pb (risk areas 1 and 2), 0.019 and 0.053 for Cr (risk areas 1 and 2), 0.028 for Ni (risk area 2), 0.045 for Mn (risk area 2), 0.007 and 0.008 for Hg (risk areas 1 and 2), and 0.01 for As (risk area 2). Cd was below LOD in all milk samples in risk areas 1 and 2.

**Fig. 5:**
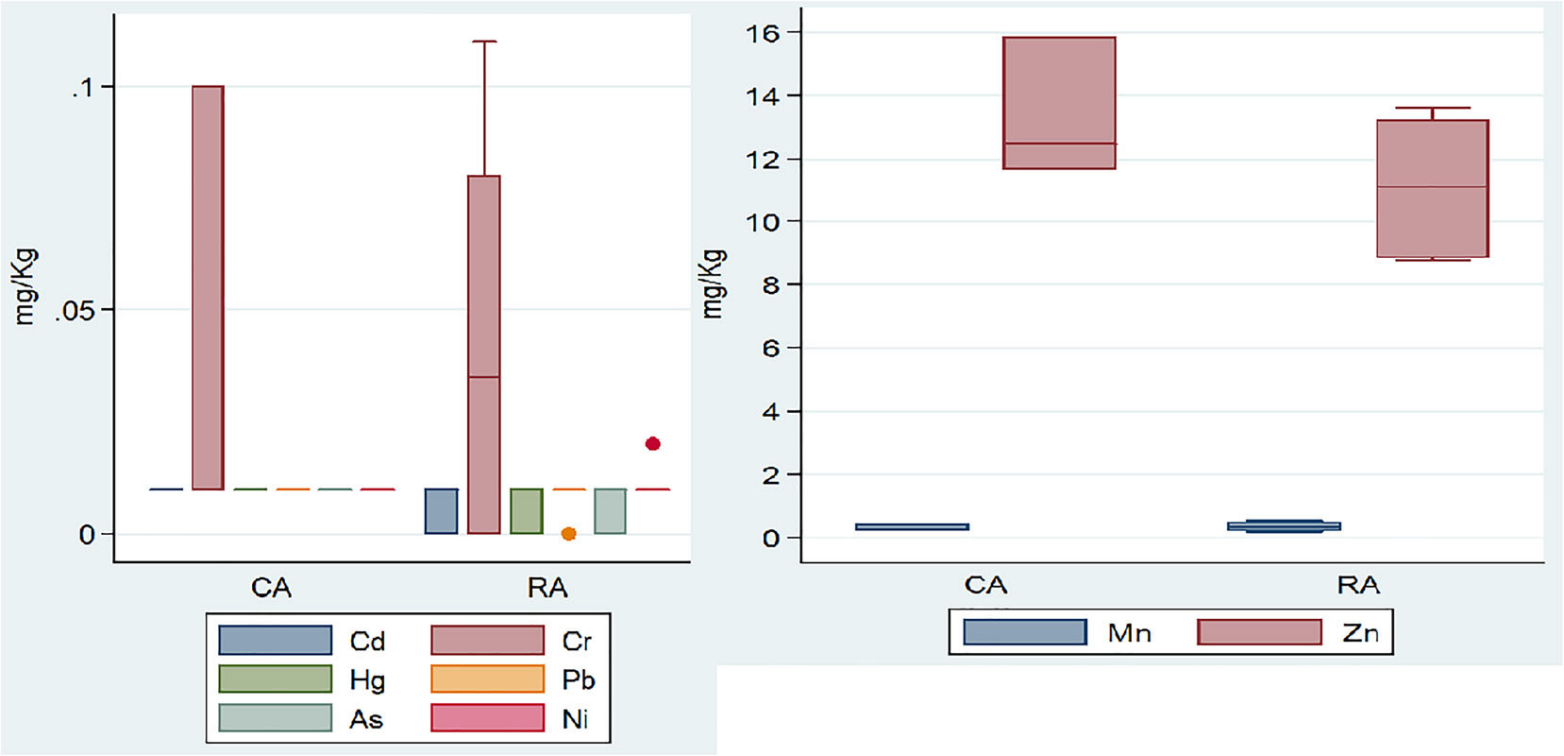
Concentration of heavy metals in eggs from control area (CA) and risk area (RA). Data are restricted to study area 2, Modena (Emilia Romagna), where they were available

Heavy metal comparison between control and risk areas was possible only for eggs in study area 2 (3 and 6 samples respectively in control and risk) given the availability of data for both areas: no significant difference was found (Fig. 5).

**Fig. 6:**
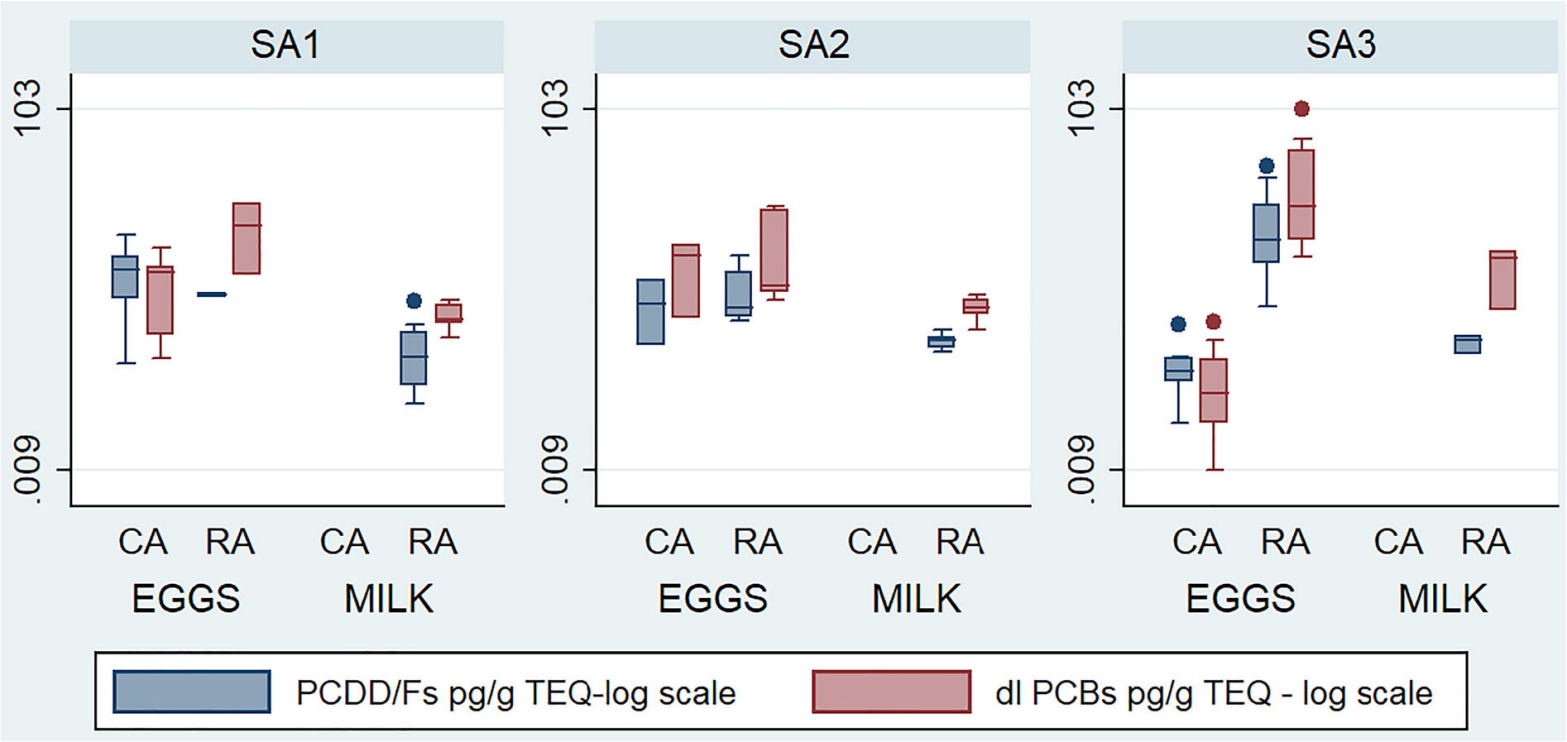
Concentration of PCDD/Fs and dl-PCBs in free-range eggs and in milk samples from the three study areas. Comparison between control (CA) and risk areas (RA)

Concentration of POP’s in milk and eggs from different areas is shown in Fig. 6.

Also for POP’s, the comparison (Step 9) between risk and control areas was possible only through eggs. The differences between the mean concentrations for POP’s were not statistically significant in study areas 1 and 2. In the study area 3 (aluminum smelter), the mean concentration in eggs was higher in the risk area for PCDD/F, PCDD/F/dl-PCB, and dl-PCB (*p* < 0.05). Moreover, considering the 5-km buffer, the linear regression model showed a statistically significant inverse association with the distance for both PCDD/Fs and dl-PCBs, clearly indicating that the concentration of pollutants in eggs decreases as the sampling distance from source increases. Based on the regression output, a 22% and 30% decrease per kilometer of distance from the source resulted respectively for the egg concentration of PCDD/Fs and dl-PCBs.

According to protocol Step 9, in case of single positivity, veterinarians inspected the farms with the aid of the standardized questionnaire to exclude the role of secondary sources of local contamination that may have acted as confounders (Step 8). For instance, in study area 1, where two rural farms showed single positive outcomes in eggs, in one of them, plastic material originally used for hens’ recovery was found scattered throughout the backyard and in close proximity of troughs; the second one was adjacent to a high-traffic road with the hens being free to wander and peck in the ditches.

## Discussion

The main outcome of the project is a standardized methodological approach for risk-based animal biomonitoring, useful in areas at risk of environmental pollution.

Thanks to the bioconcentration and bioaccumulation in animal tissues, biomonitoring is valuable in the early detection of environmental pollution and in demonstrating the involvement of the food chain and, potentially, the impact on public health, even when pollutants in soils or in crops have not exceeded the limits laid down in legislation.

Where environmental monitoring data from soil and water are incomplete or not available, animal biomonitoring can be used to fill up such a gap: for instance, pre-existent official monitoring activities on animal food (e.g., residue monitoring in food) can be easily reoriented to this purpose. Where environmental monitoring data are available and of concern, animal biomonitoring could be used before considering the opportunity of any human biomonitoring, therefore, without invoking complex ethical issues. As an added value, in this case, the concentration measured in the animal matrix makes available a quantitative proxy of human exposure, helpful to inform risk assessment. Finally, in polluted areas, the animal-based data can be used to monitor the effectiveness of remediation programs aimed at reducing human exposure.

To our knowledge, this is the first proposal for a standardized protocol for farm animal biomonitoring that can be used for both environmental risk assessment and human exposure preliminary assessment. The general methodology consists of a conceptual framework model aiming at defining sampling procedures flexible enough to be adjusted to local conditions and helpful to identify possible risks associated to a potential source by means of a target animal population used as an environmental sentinel. The 9 steps proposed here are based on good practices that were tested on field and organized in a subsequent logical way.

In the past, several animal monitoring plans have been performed, e.g., based on random sampling (EU Recommendation 2013/711) or on convenience sampling (Jafari et al. 2008) or on geographical proximity to a source of contaminants (Brambilla et al. 2011; Ingelido et al. 2009). The latter criterium was adopted also by the Italian Ministry of Health that issued a national monitoring plan for the detection of contaminants based on sampling of animal products in officially ascertained contaminated sites (Ministry of Health 2011). The plan targeted small ruminant milk and clams in marine areas (specific instructions on distance from the source, sample size, and pre-determined sampling frequency were provided). It was designed by the competent authority in order to gain preliminary information about the safety of animal products obtained from contaminated areas.

The protocol presented in this paper fits the purpose of assessing exposure to a specific putative source of pollutants whenever risk-based surveillance is to be planned (Scaramozzino et al. 2010). At the same time, flexibility to adapt to different livestock environmental settings was pursued. A “one size fits all” framework model for animal biomonitoring is not possible and each specific context may vary depending on type of pollution source, known or suspected type of contaminants, animal species and production, available resources, and social and economic acceptability. In relation to the latter issue, it is important to recall the extremely high cost of dioxin analysis and the absence of biochemical markers of mammal tissue contamination, even if recently, biomarkers for PCB’s in cows were proposed (Girolami et al. 2018). Therefore, it is of paramount importance the adoption of a sampling protocol that maximizes the results and it is cost-effective at the same time. To this end, other authors used also the pooling of samples (Adamse et al. 2017).

In the absence of evidence of flagrant contamination, for example, during a risk assessment around an emitting source, the choice of tracking contaminants or indicator molecules is crucial. This could be done on the basis of scientific literature, or from data reported from similar environmental pollution settings. In this study, we selected POP’s and heavy metals, but we are aware that other pollutants could have been present. Moreover, basically for cost-saving, we were not able to sample the same target matrix in the control and risk areas.

With regards to metals, Pb and Hg are the only metals whose maximum limit is fixed in milk by the EU legislation (EU Reg. 1005/2015; EU Reg. 73/2018). According to our results, the excess of these limits is rare even in polluted areas. The Pb mean values recorded in this study are also in accordance with the recommendation of EFSA (2010) (0.0117 mg/kg upper bound (UB) mean). Hg values were all compliant with the criteria of 50% expanded uncertainty measurement respect to maximum levels (SANTE/11813/2017). Both Cr and Ni mean values were in accordance with the EFSA recommendations (i.e., respectively 0.055 mg/kg UB for CR (EFSA 2014) and 0.031 mg/kg UB for Ni (EFSA 2015)). Mn and Zn mean values were higher than those reported in cow milk by Zhou et al. (2017) but in agreement with those measured in Arianejad et al. (2015). Therefore, there was no reason to raise any alarm to the competent authorities.

With regards to POP’s, with the exception of two milk samples above the action levels (laid down in legislation to identify the cases where it is appropriate to investigate the source of contamination and take mitigation measures) for dl-PCBs, in the risk area surrounding the aluminum smelter, all milk samples were compliant with both the maximum levels established by Commission Regulation 1881/2006, 1259/2011, and the action levels defined in the Recommendation 2013/711/UE for PCDD/Fs and dl-PCBs. PCDD/Fs and dl-PCBs exceeded maximum levels in eggs from all risk areas, while in risk areas 1 and 2, a single farm was contaminated; in risk area 3, the contamination was more spread, with a clear spatial gradient originating from the source. Eggs sampled in control areas 1 and 2 both exceeded maximum and action levels, whereas in control area 3, all the egg samples were compliant.

In case study 1, the monitoring of the risk area did not reveal any contamination by POP’s attributable to the source under investigation. Vice versa, contamination by dl-PCBs, attributable to a local source, was detected through the follow-up investigation carried out in the corresponding control area. In case study 2, eggs were found positive for dl-PCB in both the control and risk areas and the potential for on-farm secondary sources (waste incineration, storage of percolating chemicals near animal feed, proximity to roads with heavy traffic, and asphalt presence on grazing soil) was further investigated.

Findings from the biomonitoring based on free-range eggs for PCDD/Fs and dl-PCBs need to be interpreted with caution as eggs are highly sensitive to on-farm pollution (Adamse et al. 2017; Kuzukiran et al. 2018). In the presence of on-farm secondary sources, the animal contamination could not be exclusively attributed to the contaminated site and these farms should be excluded from sampling. If the secondary source is identified after the analytical activities, as a result of the questionnaire-based investigation (ESM 1), these confounding factors should be taken into account. The same attention should be paid if the animal diet is not completely of local origin. In our case studies, where egg contamination had been detected, the involved farms were subjected to a follow-up inspection by official veterinarians and a ban on egg consumption.

In our experience, the biomonitoring of dairy animals and free-range hens based on a standardized methodological approach (the 9-step protocol) provided comforting data on the human exposure in the targeted areas; the need for specific actions to mitigate the risk would have been raised if alarming contamination had been detected. In a “one health” perspective, the development and application of good practices for animal biomonitoring, made available to veterinary professionals, will promote a truly integrated approach when public health professionals are involved in environmental health assessments.

## Conclusions

This study represents the first attempt to establish standard criteria for the conduct of effective animal biomonitoring, both in areas where environmental contamination has been established and in those where it has only been suspected. As it is not possible to define a single sampling scheme valid for all situations, our procedure shows how to adapt it to the real local context, considering the characteristics of the environment involved and the source of pollution, the type of animals reared, and the socio-economic conditions. Based on the analytical results of the animal biomonitoring, local authorities can decide whether to continue the investigations by carrying out biomonitoring in humans or, alternatively, to reassure the population about the perceived risks. The application of a standardized approach prevents wastage of resources and helps to achieve an adequate sensitivity of animal biomonitoring useful both for early warning and for monitoring the effectiveness of risk mitigation measures.

## Electronic supplementary material

ESM 1 (DOC 113 kb)

ESM 2 (XLSX 26 kb)

## Footnotes

1 (http://www.who.int/hia/about/why/en/)

2 Among POP’s, 210 polychlorinated aromatic chemical compounds are described which are divided into polychlorinated dibenzo-para-dioxins (PCDDs or dioxins) and polychlorinated dibenzo-para-furans (PCDFs or furans). In this paper, the analytical activity on dioxins was restricted to the 17 congeners that are reported to have a potential health effect. With regards to the polychlorinated biphenyls (PCB), we considered only the 12 coplanar congeners that, because of their chemical structure and biological activity, have toxic properties similar to dioxins and furans and are considered to be “dioxin-like” (dl-PCBs).
