# Supplementary material for: Application of a risk-based standardized animal biomonitoring approach to contaminated sites
Source: Environ Monit Assess. 2019 Jul 30;191(8):526. doi: 10.1007/s10661-019-7653-3 (PMC6667426; doi:10.1007/s10661-019-7653-3)
Supplement: Supplementary file 1 — (DOC 113 kb) [file 10661_2019_7653_MOESM1_ESM.doc]

Article title: **Application of a risk based standardized animal biomonitoring approach to contaminated sites**

Journal name: Environmental monitoring and assessment

Author names Paola Scaramozzino^1^, Sabrina Battisti^1^, Rosanna Desiato^2^, Marco Tamba^3^, Giorgio Fedrizzi^3^, Alessandro Ubaldi^1^, Bruno Neri^1^, Maria Cesarina Abete^2^, Giuseppe Ru^2^

Affiliation and e-mail address of the corresponding author: Istituto Zooprofilattico Sperimentale del Lazio e della Toscana M. Aleandri, Via Appia Nuova, 1411 rome, Italy [paola.scaramozzino@izslt.it](mailto:paola.scaramozzino@izslt.it)

Appendix 1


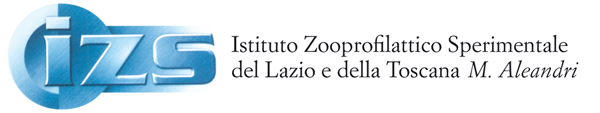


**Environmental form for on farm risk factors**

Local Health Authority__________ District______________________ Date ...../...../...….

**Farm identification code**   

Farm name ………………….................... Municipality …………....

## Geographical coordinates (WGS84 coordinate system, degrees): *

## NORTH LAT _________________________ EAST LONG __________________________

*(** *please indicate if not present or not available in the National livestock register)*

REFER TO THE LAST 12 MONTHS

**Please describe your practices of waste disposal:**

Waste delivery off-site: YES  NO  PARTIALLY 

- If YES or PARTIALLY, indicate the frequency (1=very often, 2=often, 3=sometimes)
- Delivery to incinerator 
- Delivery to public dump 
- Through municipal waste collection system 

Waste disposal on farm YES  NO  PARTIALLY 

- If YES OR PARTIALLY, indicate the frequency (1=very often, 2=often, 3=sometimes)
- Landfill 
- Farm incinerator 
- Dunghill 
- Other (please, specify) ___________________ 

**How are disposed the following materials on farm?**

**Incineration Landfill**

- PVC Materials YES  NO 
- Plastic bags of fertilizers/feed YES  NO 
- Paper bags of fertilizers/feed YES  NO 
- Rope of hay bales YES  NO 
- Boxes of drugs/disinfectants YES  NO 
- Other (describe)___________________________ YES  NO 

**In case of incineration on farm, please indicate which materials are burned**

- PVC Materials YES  NO 
- Plastic bags of fertilizers/feed YES  NO 
- Paper bags of fertilizers/feed YES  NO 
- Rope of hay bale YES  NO 
- Boxes drugs/disinfectants YES  NO 
- Other (describe)___________________________

**In case of incineration on farm, please indicate the fate of ashes:**

- They remain in place YES  NO 
- They are used for soil fertilization YES  NO 
- They are used as animal bedding YES  NO 
- They are disposed outside the farm lands YES  NO 

**Which other materials are:**

**buried in the farm landfill or disposed into dunghill?**

___________________________ ___________________________

___________________________ ___________________________

___________________________ ___________________________

___________________________ ___________________________

**Herbicides use:**

Please list the most three used (trade brand name and manufacturer)

- (1) ________________________
- (2) ________________________
- (3) ________________________
- Quantity (q/hectare) ____________________________
- Frequency (number of applications per year) ____________________________
- Where ____________________________
- In fodder fields YES  NO 
- In silage fields YES  NO 
- In grain fields YES  NO 
- In other areas where animals may have access YES  NO 

**Environmental disinfectants used**

- Which (please list the most three used)

|  | Name 1 | Name 2 | Name 3 |
| --- | --- | --- | --- |
| brand name and manufacturer |  |  |  |
| Quantity (litres per year) |  |  |  |
| Frequency (number of applications per year) |  |  |  |
| Where (which areas) | Stable/hen house YES  NO   Outside fences YES  NO   Milking parlour YES  NO  | Stable/hen house YES  NO   Outside fences YES NO   Milking parlour YES NO  | Stable/hen house YES  NO   Outside fences YES  NO   Milking parlour YES NO  |

**Insecticides use/pesticides:**

- on animals YES  NO 
- which species poultry YES  NO  sheep/goats YES  NO 
- Other species (specify) ________________
- where indoor YES  NO  outdoor YES  NO 
- which (indicate brand name) ______________________________
- Quantity (litres/year) ______________________________
- Frequency (number of applications/year) ______________________________

**Presence of asphalt/bitumen**

- In the courtyard YES  NO 
- In the access routes YES  NO 
- In animal shelters YES  NO 

Do laying hens have access to paved areas? YES  NO 

- If YES, how many hours per day? _____________________

**Problems detected in the lands bordering the farm**

- Illegal landfills. YES  NO 
- Percolation of drains YES  NO 
- Dumping of wastes YES  NO 
- Waste incineration YES  NO 
- Steel plants YES  NO 

**Animals have access to:**

- Farm garage YES  NO 
- Paints or painted walls YES  NO 
- Exhausted oils YES  NO 
- Wreckages YES  NO 
- Transformers or electric batteries YES  NO 
- Plastic materials YES  NO 

**Farm heating system (including the living area if it is the case):**

- Wood-fired YES  NO  if YES quintals/year ________
- Pellet YES  NO  if YES quintals/year ________
- LPG YES  NO 
- methane YES  NO 
- other (specify) ____________________
